# Fluoroquinolone-Mediated Tendinopathy and Tendon Rupture

**DOI:** 10.3390/ph18020184

**Published:** 2025-01-30

**Authors:** Ezgi Duman, Sigrid Müller-Deubert, Girish Pattappa, Ioannis Stratos, Stephan A. Sieber, Hauke Clausen-Schaumann, Victoria Sarafian, Chisa Shukunami, Maximilian Rudert, Denitsa Docheva

**Affiliations:** 1 Department of Musculoskeletal Tissue Regeneration, Orthopaedic Hospital König-Ludwig-Haus, University of Würzburg, 97070 Würzburg, Germany; sigrid.mueller-deubert@uni-wuerzburg.de (S.M.-D.); girish.pattappa@uni-wuerzburg.de (G.P.); 2 Department of Orthopaedics, Orthopaedic Hospital König-Ludwig-Haus, University of Würzburg, 97070 Würzburg, Germany; ioannis.stratos@klh.de (I.S.); m-rudert.klh@uni-wuerzburg.de (M.R.); 3 Center for Functional Protein Assemblies, Department of Bioscience, TUM School of Natural Sciences, Technical University of Munich, 85748 Garching, Germany; stephan.sieber@tum.de; 4 Center for Applied Tissue Engineering and Regenerative Medicine (CANTER), University of Applied Sciences, 80335 Munich, Germany; hauke.clausen-schaumann@hm.edu; 5 Department of Medical Biology, Medical University of Plovdiv, 4000 Plovdiv, Bulgaria; sarafian@abv.bg; 6 Research Institute, Medical University of Plovdiv, 4000 Plovdiv, Bulgaria; 7 Department of Molecular Biology and Biochemistry, Division of Dental Sciences, Graduate School of Biomedical and Health Sciences, Hiroshima University, Hiroshima 734-8553, Japan; shukunam@hiroshima-u.ac.jp

**Keywords:** fluoroquinolones, tendon injury, tendinopathy, tenocytes, tendon stem/progenitor cells, macrophages, endothelial cells and neurons

## Abstract

The fluoroquinolone (FQ) class of antibiotics includes the world’s most prescribed antibiotics such as ciprofloxacin, levofloxacin, and ofloxacin that are known for their low bacterial resistance. This is despite their potential to trigger severe side effects, such as myopathy, hearing loss, tendinopathy, and tendon rupture. Thus, healthcare organizations around the world have recommended limiting the prescription of FQs. Tendinopathy is a common name for maladies that cause pain and degeneration in the tendon tissue, which can result in tendon rupture. Whilst there are several identified effects of FQ on tendons, the exact molecular mechanisms behind FQ-mediated tendon rupture are unclear. Previous research studies indicated that FQ-mediated tendinopathy and tendon rupture can be induced by changes in gene expression, metabolism, and function of tendon resident cells, thus leading to alterations in the extracellular matrix. Hence, this review begins with an update on FQs, their mode of action, and their known side effects, as well as summary information on tendon tissue structure and cellular content. Next, how FQs affect the tendon tissue and trigger tendinopathy and tendon rupture is explored in detail. Lastly, possible preventative measures and promising areas for future research are also discussed. Specifically, follow-up studies should focus on understanding the FQ-mediated tendon changes in a more complex manner and integrating in vitro with in vivo models. With respect to in vitro systems, the field should move towards three-dimensional models that reflect the cellular diversity found in the tissue.

## 1. Introduction

### 1.1. Foreword

Fluoroquinolone-associated disability (FQAD) encompasses a multitude of adverse side effects of fluoroquinolones (FQs) ranging from neuropathy and ocular degradation to tendon rupture. FQADs decrease quality of life and imposes a high economic burden. This eventually led the United States Food and Drug Administration (FDA) and European Medicines Agency (EMA) to warn against the general use of FQs. Moreover, several FQs were banned because of their possible severe adverse reactions, such as gatifloxacin due to its toxicity against the nervous system [1]. Nevertheless, even FQs that show relatively low toxicity against the central nervous system, including ciprofloxacin (CPX) and levofloxacin (LFX), can cause debilitating side effects [2]. For example, CPX and LFX are associated with FQ-mediated encephalopathy, which can be observed even after 2 days of treatment [3]. Peripheral neuropathy risk also rises with FQ use. A study determined that each additional day of oral FQ use leads to a 3% higher chance of developing peripheral neuropathy [4]. LFX has been linked to delirium, while CPX in particular has a Boxed Warning against possible development of peripheral neuropathy and central nervous system side effects [3]. Despite this, FQs need to be prescribed in certain cases where other antibiotics cannot be used due to reasons such as, failed treatment or side effects [5]. Additionally, resistance against this class of antibiotics is weaker compared to β-lactam antibiotics or penicillin. Consequently, these antibiotics are prescribed regularly despite their associated adverse effects. Therefore, it is crucial to develop safer FQs or implement preventive measures to mitigate against the potential ramifications of FQ use.

**Figure 1:**
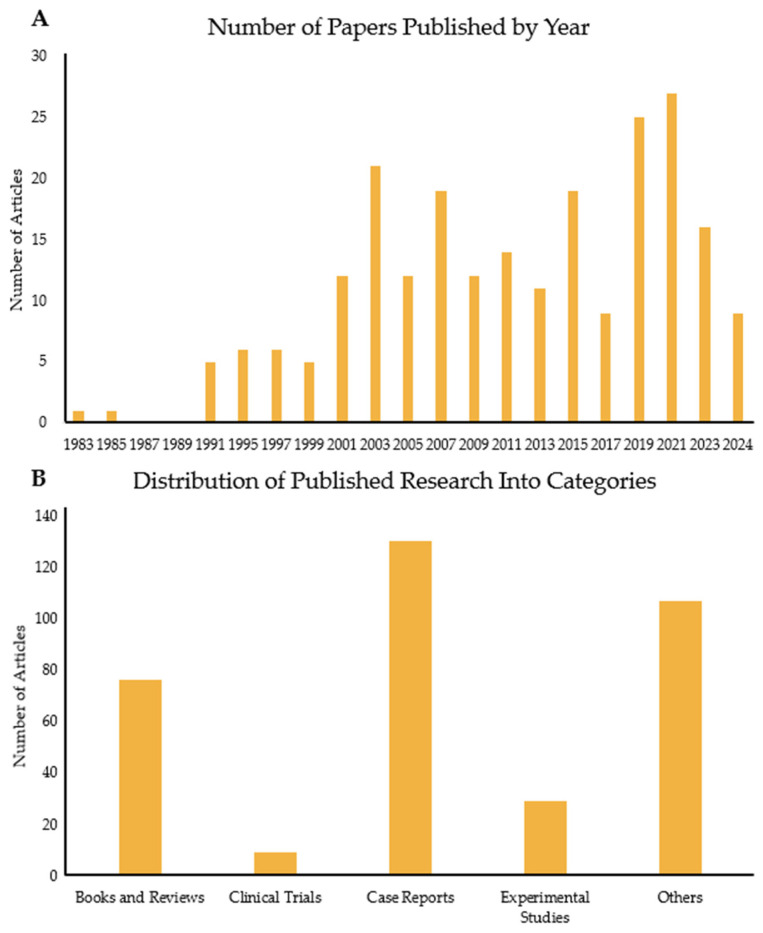
Analysis of papers published over the years on the link between FQs and tendons. (**A**) Number of papers published every 2 years from 1983 to 2024. (**B**) Number of articles published in the same period but divided into five categories: books and reviews; clinical trials; case reports; experimental studies; and others, which include the remaining publication types such as letters, guidelines, and addendums.

**Figure 2:**
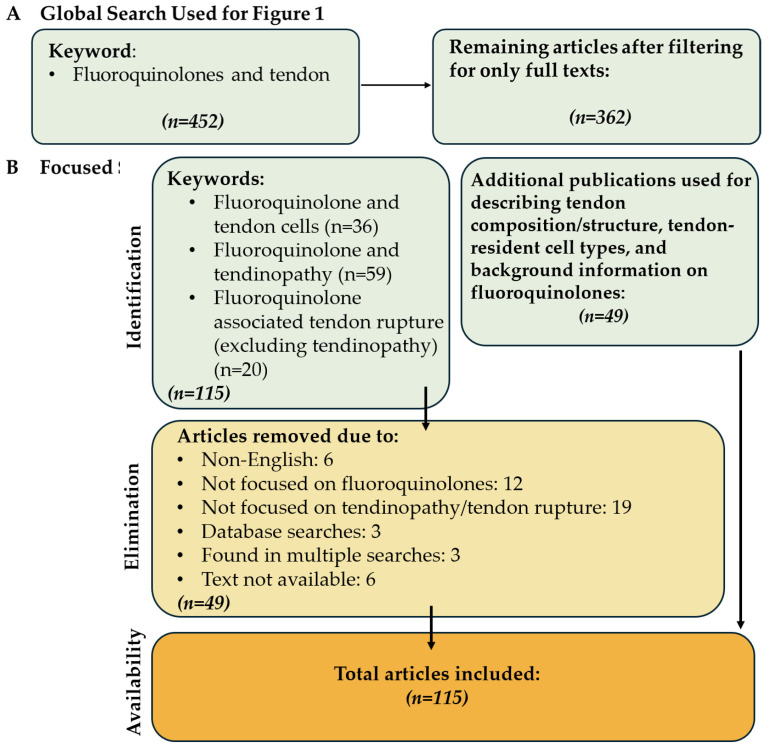
Identification and selection procedure of the articles in this review. (**A**) Articles used to construct Figure 1 and (**B**) the filtering process of the articles used in the review.

FQADs, particularly FQ-mediated tendon rupture, have received more attention over time, as the use of FQs has been linked to side effects observed in patients. Consequently, the number of published papers on this topic has risen significantly (Figure 1 and Figure 2A). Figure 1A shows the number of articles related to the search terms “Fluoroquinolones” and “tendon” over the years. The first paper mentioning both topics was published in 1983. Since then, interest in the subject has increased, with 2021 reaching the highest number of publications. Tracking this trend is important, as it reflects the growing awareness among experts about the association between FQs and adverse reactions in tendons.

Figure 1B illustrates the papers published depending on type. Most published articles are case reports, indicating the clinical problem clearly. This is followed by reviews, books, and experimental studies. The case reports are mostly on Achilles tendon rupture of older patients or those with previous morbidities, such as chronic illnesses. There are very few clinical trials, with two manuscripts targeting horses. In these tests, the effects of long-term enrofloxacin (ENR) use on adult horses or pregnant horses and their foals were assessed. In the human trials, the most tested FQ was LFX. The aim of these studies was either to assess the efficacy of LFX against upper respiratory infectious diseases (e.g., tuberculosis) or determine its safety in specific groups, such as liver transplant patients and children. One study investigated LFX as a supplementary medication against myeloma and showed that there was a significant improvement against patient symptoms when given to newly diagnosed myeloma patients [6]. Most of the studies observed higher instances of tendon disorders, although, in a comparative experiment with clarithromycin, the FQ, gatifloxacin, was found to cause no side effects [7]. One clinical trial focused on the safety of LFX use by children and investigated whether LFX causes increased rates of musculoskeletal disorders [8]. First, the potency of LFX was tested. Then, a long-term surveillance was conducted for a year, in which children were administered either LFX or another non-FQ antibiotic. The incidence of tendinopathy and other tendon disorders or associated pathologies (e.g., arthritis) was higher in children medicated with LFX compared to those treated with non-FQ antibiotics [8]. The limited number of experimental studies and clinical trials versus case reports demonstrates a clear knowledge gap between clinical reality and the mechanistic understanding of FQAD concerning tendon tissue.

### 1.2. Search Strategy

For this analysis, the advanced search function of the PubMed database was used in May 2024 (Figure 2B). Case reports were excluded from the focused search. The queries were “Fluoroquinolone associated tendinopathy”, “(Fluoroquinolone associated tendon rupture) NOT (tendinopathy)” in order to isolate papers only focusing on tendon rupture, and “Fluoroquinolones and tendon cells”. A total of 115 papers were found, excluding 49 papers that did not focus on tendon rupture/tendinopathy or were non-English, resulting in 66 papers included in this review. Additionally, 49 papers were referenced to describe tendon structure, tendon resident cell types, and FQs. As a result, a total of 115 papers were included in this review (Figure 2).

This review aims to explore the connection between FQ treatment and the development of tendinopathy or tendon rupture. The main goals were to introduce FQs, tendon structure, tendon resident cell types, tendinopathy, and tendon rupture. Further goals were to present the current understanding of how FQs impact both tenocytes and other types of tendon resident cells and to identify potential preventive measures against tendinopathy. This review also highlights promising research areas that need further investigation to decipher the causes of FQ-mediated tendinopathy and tendon rupture.

## 2. Fluoroquinolones

### 2.1. General Information

FQs are a commonly used class of antibiotics to treat diseases caused by both Gram-positive and Gram-negative bacteria. Due to their broad spectrum of activity, this class of antibiotics can be utilized to treat several different infectious conditions, including sexually transmitted diseases, urinary and respiratory infections, as well as soft tissue infections [9]. In both bacteria types, the FQs target the replication pathway by blocking DNA topoisomerase IV in Gram-negative bacteria and DNA gyrase in Gram-positive bacteria, thus effectively preventing the opening of the bacterial DNA supercoil. As such, FQs are effective against multiresistant bacteria due to their mechanism differing from other antibiotics [10]. FQs can also be internalized by eukaryotic cell types such as macrophages and fibroblasts, which allows for the elimination of intracellular bacteria but also allows the cells to act as reservoirs. For example, gingival fibroblasts (cells found in the ligaments connecting gums and teeth) can release CPX as the concentration decreases in the extracellular milieu [11]. Another reason for high FQ prescription rates is their relatively large patient age span. FQs can be given to patients of most age groups; more research is necessary to validate the safety of FQs use on neonates [12]. Additionally, it is recommended to limit the prescription of FQs in children due to the increased occurrence of arthropathy. Consequently, the application is limited to cases where there is no other applicable treatment, such as infection with multiclass-resistant bacteria [13].

**Figure 3:**
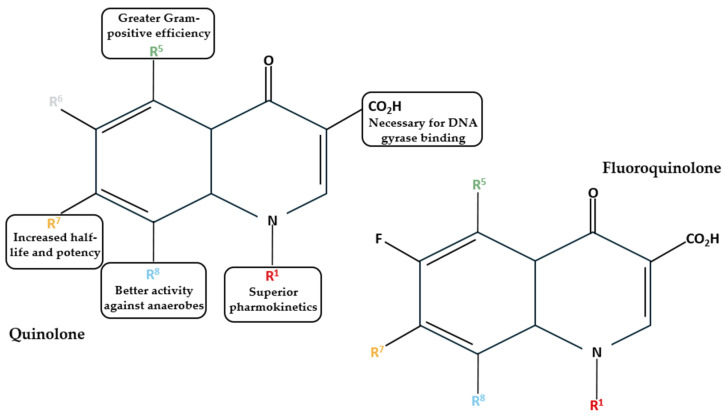
Structures of quinolones and FQs and the effects of substitute groups in six positions [1,14].

Based on their development timeline and their characteristics, FQs can be categorized into first, second, third, and fourth generations (Table 1). First generations, such as nalidixic acid and oxolinic acid, were developed in the 1960s and 1970s. This generation is also termed quinolones [14]. These antibiotics are only effective against Gram-negative bacteria and are used to treat urinary tract infections [15]. Quinolones lack the broad spectrum of activity that FQs are known for, as they lack the fluorine atom in the sixth position of the quinolone ring (Figure 3) [16]. The introduction of a fluorine atom makes the molecule electronegative and allows strong and polarized interactions between the fluorine atom of the quinolone and the carbon atom of the target [16].

Consequently, the FQs are less susceptible to microbial degradation [1]. As such, with the introduction of second-generation FQs, starting in the 1980s, many diseases could be reliably treated via oral administration [15]. Norfloxacin (NOR) was the first broad-spectrum FQ that was developed. Even so, it had lower tissue penetration and was ineffective against Gram-positive bacteria. Through the introduction of a piperazine group, the efficacy against Gram-positive bacteria was enhanced in antibiotics such as CPX, pefloxacin (PFX), and fleroxacin (FLX) [17]. Additionally, second-generation FQs also had improved cellular uptake, resulting in better intracellular clearance of pathogens [18]. However, antibiotics, such as ofloxacin (OFX), which are potent against both Gram-negative and Gram-positive bacteria, still lacked effectiveness against *Streptococcus pneumonia* [1]. This problem was improved in the third-generation FQs, and the clearance of intracellular bacteria was further refined. Interestingly, the FQs introduced in this generation are less active compared to CPX, which remains the most used FQ. Some of the most well-known members of the third generation are LFX and sparfloxacin (SPX) [1]. In the fourth generation, the overall activity was further amplified, allowing reliable targeting of anaerobic bacteria [19]. However, some of the FQs of this group have been discontinued due to severe side effects on the central nervous system [1,17]. When comparing their cellular accumulation rates in gingival fibroblasts, CPX was the least accumulated antibiotic, whilst moxifloxacin (MOX) was the highest accumulating FQ. One thing to note is that FQs are much less potent against intracellular bacteria than extracellular bacteria [19]. This can be due to multiple factors, such as weakened intracellular efficacy caused by the cellular environment and intracellular bacteria being more FQ-resistant [19]. Due to their smaller size, antibiotics such as SPX can be transported via passive diffusion into the cells [20]. The internalized FQs can then be released extracellularly via efflux transporters, which are capable of actively transporting specific compounds out of the cell in an ATP-dependent manner. For example, cultivation of the J774 mouse macrophage cell line with CPX for a long period caused overexpression of multidrug resistance protein 4 (Mrp4), an efflux transporter that can result in lower CPX accumulation in macrophages [21]. CPX was the least susceptible to being transported by efflux transporters, while MOX had the highest affinity for them [22].

Overall, the range of bacteria that FQs can target was improved over time. Furthermore, the safety of FQs was also refined consistently as new generations of FQs have been developed; however, despite the improvements, some severe side effects persist.

### 2.2. Mode of Action and Implementation

The mechanism of FQs is largely consistent across different types, despite variations in the groups attached to the main compound. All FQs selectively inhibit enzymes related to DNA replication that are not present in humans, causing them to be only active against bacteria without affecting human cells. The targeted factors are DNA gyrase and DNA topoisomerase IV, both of which are essential for normal DNA replication [23]. DNA gyrase opens the DNA supercoil, allowing the replication factors to access the DNA. DNA topoisomerase IV is responsible for the decatenation, or separation, of the intertwined chromosomes during DNA replication, ensuring that each sister cell has proper DNA segregation during bacterial reproduction [23].

In general, the treatment plans for FQs are mostly similar (Table 2). These drugs are often used either orally or intravenously depending upon the infection [24]. As most FQs have elimination half-lives that range from 4 to 10 h, these antibiotics can be taken up to three times a day [25]. For example, CPX is often prescribed for one week to two weeks at once or twice per day, depending upon the severity of the infection [26]. Third-generation FQs can even be administered once daily due to their increased efficiency [18].

The dosage can range from 200 mg to 750 mg per day [27]. The peak plasma concentration can vary from 0.6 mg/L (SPX) to 5.2 mg/L (LFX) [25]. After uptake, this peak can be achieved in two hours at the latest, although, due to their chelation characteristic, divalent cations might hinder absorption, resulting in reduced potency [27]. Elimination of FQs can occur renally, hepatically, or through the intestines. For example, PFX is cleared mainly through the liver, while CPX, ENR, and FLX can be eliminated both renally and hepatically [28]. The renal pathway can make up 76% of the total body clearance [29]. Consequently, patients with renal failure are at a higher risk for developing FQ-mediated tendon disorders due to impaired drug clearance [25].

The enhanced awareness of FQ toxicity resulted in several warnings issued over the years by multiple agencies to limit their prescription. In 2008, the United States required manufacturers to alert physicians and patients of tendon rupture and tendinitis risk. Additionally, inclusion of a Black Box warning, the highest level of warning possible, was required [30]. In 2016, this warning was expanded also to include musculoskeletal effects in combination with neuropathy and potential long-term toxicity [30,31]. Thus, the use of FQs was heavily cautioned against unless there was no other treatment option available [30]. In 2018, the FDA required psychiatric side effects to be also inserted under the “Central Nervous System Effects” section of the warning labels. In 2008, the European Union issued a recommendation to include warnings for tendon rupture as a potential side effect of FQ use; however, there was no Black Box warning [30]. The use of FQs for the treatment of mild infections or people with certain morbidities was advised against in 2018 [32]. In 2019, it was legally agreed upon that FQs must not be prescribed against mild infections or infections that can heal on their own [30]. Due to the strict guidelines the prescription rates have sharply dropped. In the United States, there were 111 FQ prescriptions per 1000 people, which decreased by 39% in 2018 [33]. Similarly, the EMA instructions also caused fewer prescriptions. For example, a drop of 25% in FQ-based therapies was observed in the United Kingdom. Overall prescription rates vary in the European Union from 0.7 to 8 per 1000 people, depending upon the country [32].

Another factor that determines the prevalence of FQADs is the average dosage recommended by physicians. For example, while Western patients can be prescribed up to 1500 mg of FQs per day, the maximum application given to Japanese patients is, at most, 600 mg [34]. These regional variations in dosages can impact the frequency of side effects and the overall incidence of FQADs. Differences in prescribing practices across countries may be influenced by cultural factors and physicians’ expectations regarding patient adherence to antibiotic regimens. Generally, FQADs are more commonly observed in countries with higher prescription levels and dosages of FQs.

### 2.3. Side Effects

Although FQs are commonly prescribed, they can be associated with severe side effects. The first recorded adverse reaction related to FQ use occurred in 1983, when a kidney transplant patient treated with NOR for a urinary tract infection later developed Achilles tendinopathy [35]. The first tendon rupture case was observed in 1988 [9]. Other severe complications of FQ use included peripheral neuropathy, nausea, or diarrhea that were observed in almost 20% of patients. However, one of the most common issues described resulted in a four-fold increase in tendinopathy risk [36]. The expansion in rupture rates might be caused by FQs’ high affinity for accumulation in connective tissues [9]. Ninety percent of FQ-related tendinopathy cases were observed in the Achilles tendon, yet other tendons might also be affected. The rectus femoris tendon, patellar tendon, and finger and thumb flexor tendons often undergo tendinopathy as well [37]. Patients ranging from 28 to 92 years of age are susceptible to FQ-mediated tendinopathy, with the condition being more prevalent in males [38]. Though the relative toxicity of different classes of FQs varies across studies, there is a general consensus that LFX is the drug most associated with tendon rupture [39]. Whilst the prescription rates of FQs have declined due to increased awareness amongst physicians and patients regarding their potential risk, the use of these antibiotics is still necessary for certain clinical cases, such as *Legionella pneumophila* infections and allergies to β-lactam-containing antibiotics [34]. Consequently, these antibiotics remain indispensable, even though there is a possibility of disability after the treatment ends. Despite it being one of the more dangerous ramifications of FQ treatment, FQ-associated tendinopathy and tendon rupture remains relatively underexplored. Therefore, more in vitro and in vivo studies are required to elucidate the molecular and cellular mechanisms.

## 3. Tendon Tissue

### 3.1. Tendon Composition and Structure

**Figure 4:**
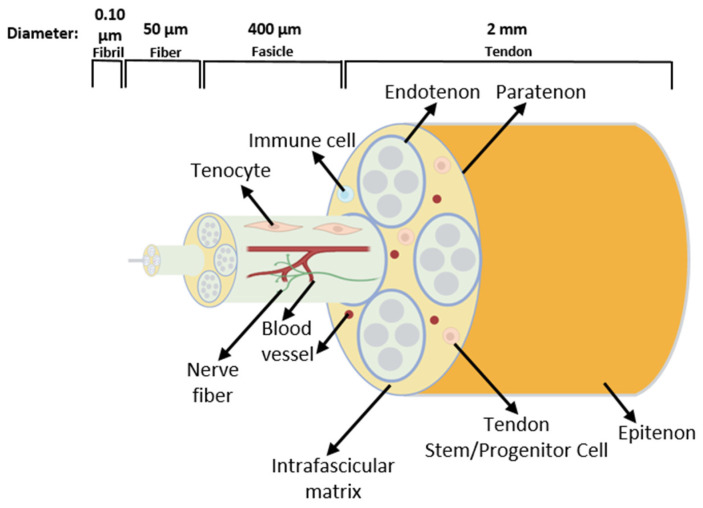
Schematic drawing of the tendon structure. The tissue consists of fascicles containing fibers built by collagen fibrils. Tendon terminally differentiated resident cells are called tenocytes. Tendon stem/progenitor cells, immune cells, blood vessels, and nerve fibers are also present in the tendon [42].

Tendons are essential structures that connect muscles with bones, allowing movement through a transfer of force generated in the muscle to the bone [40]. As in every connective tissue, the tendon cells produce large quantities of extracellular matrix (ECM). The cells form a stable structure, but this tissue has low cellular content [40]. The tendon ECM consists mainly of hierarchically structured collagen fibrils, which assemble into fibers and then into fascicles. Fascicles are covered by the endotenon and the whole tendon by the epitenon/paratenon [41] (Figure 4).

Besides collagens, the ECM contains molecules such as proteoglycans and elastin. The predominant collagen in a tendon is collagen type I, accounting for around 80% of the tendon dry weight. Since its structure is stiffer than other collagen types, it is more suitable for providing mechanical stability to the tendon and can be found in all regions of the tissue at equivalent levels. Also, collagen types II and III are present in small amounts [43]. Collagen type II is more durable compared to collagen type III; however, the diameter of the fibrils formed by this type of collagen is smaller than collagen I fibrils [44]. The most flexible of the collagen types is collagen type III, being more abundant in tissues requiring more elasticity, such as blood vessels [45]. When a tendon is damaged, at the early stages of repair, type III collagen is upregulated, and temporary scar tissue is formed to seal the tissue, which is then gradually replaced with a collagen-I-rich ECM [44]. Different types of collagens are present in varying frequencies depending upon the tissue region and status of the tendon, such as injury or inflammation. For example, type II collagen expression is higher in the tendon regions with increased compressive loads, such as the enthesis anchor point to the bone. Type I and III collagen showed no distinct spatial distribution [44]. Similar to collagen molecules, elastins are necessary for the mechanical strength of the tissue, as they form elastic fibers. Glycoproteins and proteoglycans, such as decorin, biglycan, and fibromodulin, can attach to collagen fibrils and attract water molecules. During tendon development, decorin and fibromodulin influence and regulate the formation of collagen fibrils of proper diameter and size. Moreover, their deficiency leads to alteration in the collagen fibril diameters and compromised biomechanical function of the tendon [46]. Between the collagen fibrils, there are the resident fibroblast-like cells known as tenocytes [47] that are responsible for ECM synthesis [48]. While tendon tissue was considered to contain a very limited number of cell types, this preconception has been challenged by the identification of various tenocyte subtypes and other tendon resident cell populations. These will be discussed in Section 3.2. and Section 3.3.

### 3.2. Tendon-Lineage Cells

Tenocytes differentiate from tendon progenitors that originate from mesenchymal stem cells (MSCs). The presence of certain growth/differentiation factors, such as Transforming Growth Factors (TGF)-β and Fibroblast Growth Factors (FGF), is necessary for the expression of tendon-specific transcription factors, including Scleraxis (Scx), Mohawk (Mkx), and Early Growth Response Factor 1 (EGR-1) [49]. Expression of these factors results in the transcription of other tendon-specific genes such as tenomodulin (TNMD), collagen, and Small Leucine-Rich Proteoglycans (SLRPs), the expression of which is necessary for tendon tissue development [49]. Fully differentiated tenocytes are spindle-formed with long membranous processes and aligned longitudinally [50]. However, there are various tendon cell subtypes identified by multiple studies utilizing identification methods such as RNA sequencing, flow cytometry (FACS), and quantitative RT-PCR (qPCR). These cell populations have different gene expression profiles and, thus, are cumulatively termed tendon-lineage cells (Table 3). For example, five subtypes of tenocytes are described by Kendal et al., 2020. All of these subtypes express collagen matrix genes, yet the expression levels of decorin and elastin microfibril interfacer 1 were higher in tenocyte types A and B compared to the others. Tenocyte subtype E was predominantly found in the tibialis posterior and peroneal tendons, whereas tenocyte subtype A was more prevalent in the toe extensor tendon [51]. Another study identified 10 tenocyte subsets found in either healthy or diseased human tendons, each exhibiting unique gene expression profiles and potentially distinct functions. For instance, tenocyte subset 2 was found to express more proliferative factors compared to the others, while tenocyte subset 3 expressed mainly anti-inflammatory factors [52]. With tendon aging, the tenocytes show reduced expression of factors such as Mkx, leading to a decrease in fibril diameter [49]. Mature tenocytes are characterized by limited replication activity, restricting the self-renewal capacity of the tendon tissue [53]. Nevertheless, multipotent cells with proliferative capacity are also present within the tendon and are known as tendon stem/progenitor cells (TSPCs) [54]. In adult tendons, the TSPCs play a crucial role in the tendon repair process by migrating to the injury site, where they differentiate into tenocytes. As TSPCs age, their restorative ability becomes limited due to reduced self-renewal and increased senescence that are concomitant with slower migration rates and worsened cell–ECM interactions [54]. Due to their increased senescence, decreased metabolic activity, and ECM biosynthesis, the aged TSPCs might have impaired contribution to homeostasis, which, in turn, may trigger tendinopathy [55]. How, exactly, aged TSPCs impact tissue sustainability is still not fully understood and should be further investigated. Interestingly, there are also TSPC subtypes and some might be proliferative, whilst other subsets might be more motile [56]. Moreover, certain TSPC subpopulations might be activated and become more dominant in tendinopathic tendons and contribute to exacerbation of the symptoms by secreting pro-inflammatory factors [57]. Such subsets might also have different functions in processes such as tendon repair. For example, CD146^+^ cells in the intrafasicular tendons can migrate toward sites of injury and are capable of mineralization [58]. A study also identified subsets that can secrete inflammatory factors to recruit immune cells to facilitate tendon repair or ones that are capable of enhanced collagen production and proliferation [59]. Depending upon the prevalence and the functionality of the cells, the repair process can be fibrotic due to a disorganized ECM. However, these tendons are more prone to reinjury that is caused by a loss of tendon tissue integrity [60].

Multiple studies indicate that there are tendon-lineage cells with very distinct characteristics in the tendon tissue. However, there is no consensus on the nomenclature of the identified cell subtypes. Moreover, the gene marker profiles are mostly based on mRNA profiles and the proposed functions of the cell subtypes have yet to be validated. Hence, more experimental studies are needed to build upon the recent exciting findings on the cellular heterogeneity of the tendon tissue. The current understanding of the interplay between tenocytes, TSPCs, and other cell types, such as immune cells, within the tendon tissues remains limited and investigations utilizing sorted and purified cell subsets will be of great value for gaining more precise insights. In summary, tendon tissue is far more heterogeneous than previously understood, and clarifying the cellular diversity, cell–cell communication, and regulatory mechanisms in both health and disease is of utmost importance for deciphering tendon pathology and regenerative processes, including FQ-mediated tendinopathy and tendon rupture.

### 3.3. Other Tendon Resident Cell Types

In addition to tenocyte subtypes and TSPCs, tendons also contain a small portion of adipocytes, resident immune cells, nerves, and endothelial cells that build blood vessels, which contribute to regulating the homeostasis of the tissue under physiological conditions. The main resident immune cell types of the tendon are macrophages, which belong to the innate immune system. Macrophages and other innate immune system cells are responsible for initial nonspecific responses against infections and tissue damage [71]. Furthermore, they are also important for the regulation of tissue homeostasis. In the tendon, there are two distinct macrophage subtypes: tissue-resident and blood-recruited [71]. The tissue-resident macrophages colonize the developing tissues during embryogenesis [72]. During tendon growth, the resident macrophages are located adjacent to tendon fibroblasts and are present from embryonic day 15 onwards [73]. Tendon resident macrophages can have three distinct morphologies in the tendon tissue: macrophages similar to fibroblasts, elongated macrophages, and macrophages that wrap around fibroblasts [73]. There are also tenophages, which are macrophage-like tendon cells that become activated following a tendon injury. These cells co-express macrophage markers, including CD206 and C-X3-C motif chemokine ligand 1/receptor 1 (CX3CL1/CX3CR1), along with tendon markers such as Scx [74]. Macrophages that are in close contact with fibroblasts can be regulated by receptor–ligand connections, such as interleukin (IL)-6 produced by fibroblasts, which induces inflammation, or Colony-Stimulating Factor 1 (CSF1) signaling that supports the survival of macrophages [73]. In contrast, macrophages can present regulatory factors to fibroblasts to control their proliferation and differentiation such as TGF-β1 and Platelet-Derived Growth Factor (PDGF)-B. Tendon resident macrophages express CD206 at a very high level. This type of receptor plays a crucial role in the internalization of degraded collagen, thereby facilitating ECM turnover [73]. Additionally, macrophage-derived matrix metalloproteinases (MMPs) can also enhance collagen replacement during tendon healing [75]. During tendon repair, there are several subsets of recruited macrophages, where some of them show a more inflammatory or M1-like profile, whilst others express anti-inflammatory (M2)-type markers. Several subsets express characteristics of both profiles, emphasizing the diversity of macrophages involved in the tendon repair process [66]. Although adaptive immune cells such as T cells can also be found in tendon tissue, their role remains relatively unknown. One study has determined that the T cells can express CCR2, which prevents autoimmunity and restricts inflammation. This suggests that resident T cells may be active during tissue healing [76]. Nevertheless, more data are required to accurately evaluate the role of T cells in the tendon tissue.

Another cell type found in the tendon is endothelial cells, which determine the vascularization and the homeostasis of the capillaries. Adult tendons are not highly vascularized, as they rely on synovial fluids for nutrition rather than blood [77]. However, upon tendon injury, the tendon tissue requires improved blood flow to fuel tendon repair through delivery of nutrients and enhanced invasion of immune cells. Thus, angiogenesis is an essential step in the healing process [77]. During tendinopathy, the vascularization of tendon tissue also expands during the early stages, although the exact cause of this neovascularization remains unknown [78].

Nerve fibers are also present in the healthy tendon tissue, yet they are only localized in the paratenon [79]. During tendon repair, neurons grow further into the tendon tissue to mediate the regulation of healing. Conversely, during tendinopathy, the neuronal activity is increased and most likely contributes to inflammation and a sensation of pain [79].

Non-tenocyte cells in tendon tissue are a mostly new but intriguing area of study. A better understanding of the interaction between the tendon and its resident cell types can assist in developing novel strategies for preventing tendon conditions. The investigations on the tendon resident immune cells have mostly focused on macrophages, although lymphocytes, monocytes, neutrophils, mast cells, and other immune cells should also be studied in the context of the tendon microenvironment. It is important to determine the function of these cells since they play various roles, such as clearance of debris, fighting infections, immune cell recruitment, regulating tissue homeostasis, and steering tendon repair. Therefore, any FQ-mediated disturbance in immune cells can trigger or exacerbate FQADs.

## 4. Fluoroquinolones and Tendon Diseases

### 4.1. Fluoroquinolones and Tendinopathy

Tendinopathy is an umbrella term for conditions mostly associated with pain, swelling, and impaired function of the tendon. Tendinopathies can be divided into three groups. Tendinosis is the degeneration of the tendon due to compounding damage over time without the influence of inflammation [80]. In contrast, tendinitis is tendinopathy induced by the inflammation of the tendon and the presence of inflammatory cells. Lastly, tenosynovitis is the inflammation of the membrane surrounding the tendon tissue [80]. Tendinopathy occurs in three phases. In stage one, the tendon is micro-injured, which triggers the release of pro-inflammatory factors and MMP expression increases [80,81]. In the following step, the injury cannot be repaired despite the healing. The repair can fail for a variety of reasons ranging from genetic predisposition to the use of certain medications, such as FQs. The last stage is when the clinical symptoms are evident and irreversible alterations in the tendon tissue occur. These can include increased vascularity and changes in the cell content, ECM composition, and organization [81]. Tendinosis can be organized into categories such as hypoxic, hyaline, fibrinoid, mucoid/myxoid, and fatty degeneration depending upon the phenotypic changes [82]. For example, mucoid degeneration is caused by the augmented deposition and diffusion of glycosaminoglycans into tendon fibers [83].

During tendinopathy, the tendon tissue undergoes multiple alterations, including disorganization of the ECM, thinning of collagen fibers, reduced collagen content, and, in some cases, increased tendon stiffness [80]. TSPCs also participate in the development of tendinopathy, as they can differentiate into cell lineages other than tenocytes, such as osteogenic adipogenetic or chondrogenic lineages. This induces abnormal tissue development in tendons and causes a weakening of the tissue structure [84].

Long-term tendinopathy can result in tendon rupture [85]. Other reasons for rupture can be trauma caused by accidents and degeneration of the tendon due to age. Several factors can exacerbate this process, such as corticosteroid or FQ use and diabetes [86]. Overall, tendinopathy is triggered by overuse of the tendon with cumulative structural damage occurring over time. In more severe cases, this ends with a rupture of the tendon. Certain diseases, lifestyles, or medications can promote the development of tendinopathy.

### 4.2. Fluoroquinolones Link to Tendinopathy and Tendon Rupture

The chance of developing FQ-mediated tendon rupture is relatively low when an otherwise healthy individual uses this class of antibiotics. In this case, the estimated number of patients affected by FQ-mediated tendon disease is around 20 per 100.000 patients [17]. The predisposing factors include exceeding 10.000 mg of cumulative FQ dose in a year [87], oral uptake instead of intravenous uptake, old age, and gender [88]. However, in patients with risk factors, this probability rises significantly. For example, up to 6% of tendon rupture cases in patients older than 60 can be traced back to FQs [39]. The findings are contradictory regarding which gender is more susceptible to tendon rupture after FQ treatment, with some studies suggesting that males are more likely to develop tendon injury after the FQ regimen [88]. Other papers propose that females are instead more susceptible [87]. Furthermore, each additional day of antibiotic application can increase the risk of tendon rupture by around 6% [89]. The average prescription duration is 2 weeks; though, in more severe cases, the regimen can be longer, up to around 81 days [90]. Adverse effects of FQ administration are often observed after the first week, but, in some cases, this can occur up to several months after the first prescription, even after the use has ceased [38]. Additionally, FQs can affect tendon healing negatively when taken after primary tendon repair surgery. When CPX or LFX were administered within 90 days post-operation, tendon rupture rates were around 2-fold higher compared to patients who did not use FQs [91]. While most FQs are associated with tendon injury, some are more toxic than others. LFX was the most common source of Achilles tendon rupture [88], whilst CPX, PFX, OFX, and NOR were also commonly associated with such cases [39]. This can be due to several reasons. If an FQ contains a methylpiperazinyl substituent rather than a piperazinyl group, this causes a higher chance of tendon injury [34]. Most of the antibiotics that are associated with tendon rupture belong to the second and third generations that commonly have methylpiperaznyl substitutes. However, in the third-generation FQs, this group was replaced with piperazinyl, and this correlates with a lower risk of tendon rupture [34]. Another possible reason for this malady might be the chelation of magnesium ions, which induces a decrease in type I collagen synthesis. Thus, lowered collagen production can result in tendon weakening and possibly a rupture [92]. The overall lower prevalence of FQ-mediated side effects in the healthy population might explain the lower number of experimental studies shown in Figure 1B, although the risk of FQADs remains high for certain patient cohorts such as transplant or chronic infection patients. Consequently, a better understanding of what triggers FQ-mediated tendinopathy and tendon rupture remains crucial. In general, various FQs can trigger tendon problems at differing frequencies and intensities due to their varying chemical profiles. Thus, it is important to consider which antibiotic is suitable for the infection and the patient using it.

### 4.3. Fluoroquinolones Effects on Tendon-Lineage Cells

Whilst the side effects of FQ use are common knowledge, the molecular and cellular mechanisms behind this phenomenon remain largely unknown. Research suggests that FQs activate MMP1 and MMP2, which degrade collagen type I and others. The activation of these enzymes causes an overall reduced amount and size of collagen fibrils [93]. The changes in collagen content alter the ECM, resulting in an overall deterioration of the tendon tissue, followed by rupture. Another factor which can lead to tendinopathy is the weakening of the cell–ECM matrix interactions by lowering integrin receptor expression on tenocytes [94]. CPX-mediated increase in reactive oxygen species (ROS) also results in dysfunction in the mitochondria, which, in turn, may induce tenocyte death [27]. In addition, CPX was reported to affect cell spreading and lamellipodial formation [95], as well as causing a cell cycle arrest at the G2/M phase in rat tendon cells [96].

Several studies have investigated the effects of FQs on tendon cells from different species. In general, FQ administration has been shown to negatively affect collagen production [27], increase the expression of MMPs [97], and even cause cell death [98]. The findings of several studies are summarized in Table 4.

Although these studies contribute to a better understanding of the molecular and cellular mechanisms behind the FQ-associated tendon disease, the outcomes can be contradictory. This indicates a need for more and improved in vitro models. Still, there is a clear trend of direct cytotoxicity, as well as alterations in gene expression induced by FQs in the tendon-derived cells.

### 4.4. Fluoroquinolones Effects on Immune Cells, Endothelial Cells, and Neurons

While FQs can influence tenocytes directly, recent research also points towards the involvement of other cell types in FQ-mediated tendinopathy. Due to the changes in the metabolic environment, the metabolites that regulate immune activity are altered [108]. This can lower the phagocytic rate of macrophages and prevent debris elimination. In addition to this, FQs can also affect the mitochondrial function in immune cells, leading to the inhibition of macrophage phagocytosis [108]. Furthermore, FQ use can also shift the surface transporter profile of macrophages, which can alter the cellular accumulation levels of FQs [21]. There is also evidence that FQs, even at residual concentrations (0.1 μg/L), can result in polarization of macrophages to a pro-inflammatory subtype [109]. As mentioned before, peripheral neuropathy is also a prominent side effect of FQs. This condition is defined by neuron damage, resulting in numbness and pain in the affected region. Development of neuropathy after FQ treatment is associated with inhibition of Gamma-aminobutyric acid receptors that induces decreased stimulation of neurons and increased toxicity [5]. There is little information on tendon resident endothelial cells and how they are affected by FQ. However, an in vitro study on corneal endothelial cells showed that FQs caused a dose-dependent cytotoxicity. The cytotoxic effect of CPX and OFX was higher compared to LFX [110]. Details about how FQs affect tendon resident cells other than tenocytes are extremely scarce; yet, without understanding how tendon resident cells are affected and how their crosstalk is impacted after FQ use, it is impossible to determine the complete mechanisms of FQ-mediated tendon damage. As such, it is essential to explore the FQ mode of action in more complex cell models.

### 4.5. Studies on FQ-Mediated Tendon Rupture Using In Vivo Models

Most of the research on this topic had been focused on in vitro tests due to their ease of application. However, these tests are limited in their scope, due to them not simulating the complete tendon environment. To this end, only a few in vivo studies have also investigated FQs and their effects on tendons and associated cell types, using different animals as model organisms. A study of PFX on mouse tendons using 400 mg per kg, which is a typical prescription dosage, assessed proteoglycan synthesis and collagen modifications after rotator cuff surgery [111]. These assays indicated oxidative damage of type I collagen, such as fragmentation. This paper also proved that PFX leads to a decrease in proteoglycan synthesis after 24 h [111]. Interestingly, a similar depletion of proteoglycans was also observed in cartilage, indicating the mechanisms of FQ-mediated tendinopathy and cartilage damage might be similar [111]. The damage was caused by oxidation, which was hypothesized to be caused by impaired mitochondrial activity [111]. In another experiment using rat rotator cuff repair, the rats were divided into four groups depending on the FQ treatment regimen [112]. In group one, the rats were given FLX for a week before the operation. In group two, they were given FLX one week before and two weeks after the surgery, and, in the third group, the animals were given FQs for only two weeks after the surgery. The last group did not receive any antibiotic treatment. The results demonstrated a 30-fold increase in MMP3 expression and a 7-fold increase in MMP13 levels in tendons following FLX administration in group two [112]. All groups except the control showed poorer collagen organization. After recovery, the second group could carry less weight than the others. The cross-sectional area of the tendon also decreased if the rats were given FLX before and after surgery [112]. One more study compared the toxicities of multiple FQs using rat models. The rats were orally given 100, 300, or 900 mg/kg in a fixed volume of 10 mL/kg once every day, and the Achilles tendon was isolated from both treated and untreated rats [113]. Increased lesions were observed in these tendons with characteristics such as dilated blood vessels, more mononuclear cells, or edema. Higher amount of mononuclear cells and edema were also observed in the synovial membrane close to the lesions. These tissue anomalies were also dose-dependent, with lesions becoming more severe with increasing concentrations [113]. Out of the investigated FQs, PFX and FLX were found to be most toxic (observed in concentrations 100 mg/kg upwards), and LFX and OFX were less toxic (effect seen from dosages 300 mg/kg and up). SPX-treated cells were negatively affected when given the 900 mg/kg concentration. Meanwhile, CPX and NOR caused no adverse outcomes. Inflammatory cells also infiltrated the tendon tissue, resulting in an increased inflammation of the tendon and degradation of the ECM [113]. In vivo models present a chance to explore complex mechanisms and interactions of FQs in the natural environment and, thus, it is essential to conduct more experiments with different model organisms. All in all, studies involving animal models have so far confirmed the in vitro outcomes, whereby FQ application led to an increase in collagen degradation and impaired tissue healing that ultimately leads to the development of tendinopathy and tendon rupture.

### 4.6. Preventative Measures and Treatment

Whilst FQs can lead to irreversible changes in tendon tissue, utilization of certain medications alongside FQs can reduce the chance of developing tendon illnesses. One of the most cytotoxic adverse events of FQs is increased production and accumulation of ROS in cells [114]. This ultimately causes oxidative stress due to mitochondrial membrane damage and cell cycle arrest. In addition, damage to mitochondrial DNA is also possible due to the mutagenicity of ROS [114]. As such, one of the main strategies is to administer antioxidants such as MitoQ or anethole dithiolethione along with FQs [114]. Co-administration with platelet-rich plasma can enhance the restorative capabilities, due to its protective capability against tenocyte senescence and death [115].

Vitamin E is an additional possibility, as a preventative care, due to its capability to prevent free-radical damage [9]. The application of these antioxidants can trigger unexpected adverse reactions, such as enhanced toxicity, when applied together with FQs and should therefore be considered as a last resort. For this reason, the focus should remain on developing FQs that have as little damage to the tendon tissue as possible.

## 5. Conclusions

Although FQADs are relatively rare, vulnerable populations that are at high risk include older patients and transplant recipients. Despite the heightened risk of adverse events, FQs are a common choice of antibiotics due to their efficacy and easy access. One of the more severe side effects of FQ-based infection therapy is tendinopathy and tendon rupture. This is mostly caused by ECM dysregulation, changes in collagen synthesis pathways, and alteration in tenocytes in multiple ways, such as lowered proliferation and halted cell cycle, increase in expression of collagen-degradation-associated genes, damage to the mitochondria membrane, reduced mitochondrial activity, and even deviations in cell shape and migration, indicated by both in vitro and in vivo assays. Accumulation of these disruptions of normal tendon environment and function can lead to tendon diseases. In recent years, FQ-mediated tendinopathy has gathered more interest. Though the research has focused on tenocytes and ECM of the tendon, there has been recent interest in other tendon resident cells. These cells contribute to maintaining regular homeostasis for the tendon, and their interactions with tenocytes play a role in preserving the health of the tissue. Consequently, FQ-mediated changes ranging from altered metabolic function of macrophages to neovascularization to spreading of the nerve endings can exacerbate tendon disease.

## 6. Future Directions

There remain many open questions, such as what factors FQs bind in the tendon cells, whether they accumulate in the tissue over time, and why inflammation exacerbates this. So far, most experiments on FQADs are conducted in monoculture, where only one cell type is studied in vitro. Since tendon tissue contains different types of cells, monoculture models do not capture the complexity of cell–cell communication and paracrine effects. Therefore, in future research, co-culture experiments in 2D and 3D models should be considered, in order to gain a better understanding of the tendon environment in the presence of FQs. In addition, implementing in vivo tendinopathy and tendon rupture models in combination with FQ administration could provide valuable clarification on the pathological processes that are closer to the clinical situation. Whilst certain alterations in the tendon tissue and tendon resident cells during FQ-mediated tendinopathy are already described, it is still not known in what order these events occur. Thus, an integrative approach of more complex in vitro systems closer to the clinical setting and in vivo models would be necessary to achieve a breakthrough in decoding FQAD in relation to tendons.

## Figures and Tables

**Table 1:** FQs mentioned in this study categorized into generations and their typical characteristics [1,17].

Generation	FQ	Characteristics
Generation 1	Nalidixic acid Oxolinic acid	Used for only urinary tract infections Not active against Gram-positive bacteria No anti-*Pseudomonas* activity
Generation 2	Ciprofloxacin (CPX) Enrofloxacin (ENR) Norfloxacin (NOR) Ofloxacin (OFX) Pefloxacin (PFX) Fleroxacin (FLX)	Amplified effectiveness against Gram-negative bacteria First generation that targets Gram-positive bacteria Better tissue penetration Longer half-life
Generation 3	Levofloxacin (LFX) Sparfloxacin (SPX)	Enables once-a-day dosing Reduced toxicity against the nervous system
Generation 4	Gatifloxacin Moxifloxacin (MOX)	Overall increased activity Potent against anaerobes

**Table 2:** Various bacterial infectious diseases that can be treated with CPX [26].

Bacteria	Gram +/−	Disease
*Bacillus anthracis*	+	Anthrax
*Enterococcus faecalis*	+	Urinary tract infections
*Helicobacter pylori*	−	Salmonellosis
*Klebsiella pneumoniae*	−	Pneumonia
*Legionella pneumophilia*	−	Legionnaires’ disease
*Mycobacterium tuberculosis*	+	Tuberculosis
*Neisseria gonorrhoeae*	−	Gonorrhea

Note: prescription duration: 1 to 2 weeks; dosage: 250 to 1500 mg for once or twice a day; uptake method: oral or intravenous.

**Table 3:** Cell types found in tendon and different subtypes of tendon-linage cells identified in multiple species. Studies are ordered chronologically.

Cell Types	Species	Identification Methods	Characteristics and Markers	Study
Mkx^+^ or Platelet Derived Growth Factor Receptor Alpha (PDGFRA^+^) fibroblasts	Human	Single nucleus RNA sequencing, spatial transcriptomics, immunofluorescence staining, Masson’s trichrome staining, hematoxylin, and eosin staining	• Mkx^+^ Cells: Expression of *TNMD* and thrombospondin 4. Present in the entire hamstring Markers: *MMAFB*, *ZFHX3* • PDGFRA^+^ Cells: High expression of neuronal growth regulator 1 and fibrillin. Increased elastin fiber formation. More abundant in areas closer to skeletal muscle Markers: *HOX2*, *TCF7L2*	Mimpen et al., 2024 [61]
Intrafascicular CD146^+^ tendon cells in healthy tendons	Horse	Network-based interaction predictions, immunolabeling, fluorescent labeling, 3D immunolabeling, FACS, immunohistochemistry, clonogenic, adipogenesis, and osteogenesis assays	No stem-cell-like characteristics, migration towards injury sites, capable of lipid production Markers: CD146, CD44	Marr et al., 2023 [58]
Tendon fibroblast 1 and tendon fibroblast 2	Rat	Spatial transcriptomics	• Tendon fibroblast 1: Positioned in the central tendon and are responsible for COL1A1 deposition Markers: *Comp*, *Cilp2*, *Dcn*, *Col1a1* • Tendon fibroblast 2: Located in the area near the tendon–bone connection and is responsible for the deposition of collagen that envelops the tendon Markers: *Apoe1*, *Col3a11*, *Cfd2*, *Tmsb4x2*	Steffen et al., 2023 [62]
TSPC-0 to TSPC-7	Human	Wound healing assay, migration assay, three-way differentiation capacity evaluation, transwell assay, transposase-accessible chromatin sequencing, single-cell RNA sequencing	• TSPC-0: Subtype that senses inflammation Marker: *AKR1C1* • TSPC-1: Capable of migration Markers: *STC2*, *HMGA1* • TSPC-2: Associated with fibrosis Markers: *SLIT3*, *LUM* • TSPC-3: Capable of proliferation Marker: *MK167* • TSPC-4: Expression of anti-inflammatory factors Marker: *FABP5* • TSPC-5: Dominant in tendinopathy samples, adipogenic differentiation regulators expressed Markers: *ADIRF*, *CRABP2* • TSPC-6: Important for ECM modeling Marker: *MXRA5* • TSPC-7: Lacks tendon healing-related genes and related to halted cell migration Markers: *MALAT1*, *MEG3*	Guo et al., 2023 [56]
Tenocytes 1–10 (TC1-10), macrophages, endothelial cell subpopulations 1–5	Human	Immunohistochemistry, multiplex immunofluorescence staining, single-cell RNA sequencing	• TC1-3: Dominant in healthy tendons, TC1 cells are normal fibroblasts, TC2 express high levels of ECM components and proliferation regulators, TC3 cells can act anti-inflammatory Markers: *COL1A1*, *COL3A1*, and *PDGFRB* (TC1), *MEG3*, *COL1A2* (TC2), *PLPP3*, *PLA2G2A* (TC3) • TC4 and 5: Attachment of tendons to the bones Markers: HAS1, PRG4 (TC5) *MYOC* and *IGFBP6* (TC5) • TC6: Inflammatory fibroblasts Markers: *PTGFR*, *SAA1* • TC7: Expression of genes related to tendon repair Markers: *TPP3*, *COL3A1*, and *COL5A1* • TC8-9 are chondrocytes. TC10 can be classified as osteocytes Markers: *CRLF1* and *CD55* (TC8), *SOX9*, *OGN* (TC9), *SPP1*, *IBSP* (TC10)	Fu et al., 2023 [52]
Synthetic, native, reactive, fibrotic, inflammatory, and muscle-associated adult Scx-expressing populations in post-repair tendons	Mouse	Histology, immunofluorescence, spatial RNA sequencing, pseudotemporal ordering and lineage trajectories, cell–cell interactome analysis	• Synthetic population: Involved in ECM and collagen fibril organization Markers: *Tnmd*, *Fmod*, and *Col1a1* • Native population: Found in areas distant from tissue injury Markers: Musculoskeletal markers • Reactive population: Mostly near the injury site, associated with higher collagen catabolism, and an increase in adhesion, migration, and proliferation Markers: Higher expression *Mmp13* and Lox • Fibroblastic population: High levels of ECM synthesis Markers: *Col3a1*, Postn Inflammatory population: Expression of markers more similar to immune cells such as macrophages Markers: *Saa3* and alarmins • Muscle-associated population: Characteristics Marker: *Mb*	Ackermann et al., 2022 [59]
Paratenon TSPC	Human	Histochemical staining, electron microscopy, immunostaining, immunohistochemical analysis	• Expression of smooth muscle actin Markers: CD105 and CD146	Zhang et al., 2021 [63]
High nestin (*NES*^+^) clonogenic TSPCs, Solute carrier family 40 member 1 (*SLC40A1*)^+^ TSPCs, pro-inflammatory TSPCs (piTSPC)	Human	Drop-sequencing test, 10X chromium test on stretched cells, mitotracker staining, qPCR	• piTSPCs: Most likely involved in inflammation-mediated development of tendinopathy Markers: *IL6*, *IL8*, and *CXCL1* • *SLC40A1*^+^ TSPCs: Involved in tendon development and repair due to their higher expression of TGFs Markers: *TGFB2*, *TGFB3* • *NES*^+^ clonogenic TSPC Markers: *MKI67*, *TOP2A*	Still et al., 2021 [57]
Interfascicular TSPCs	Human	Immunolabeling, confocal imaging, qPCR of cell culture serum	• Possible higher expression of CD146 and higher accumulation in injury sites in some TSPCs Marker: CD146	Marr et al., 2021 [64]
Tenocytes A–E present in diseased and healthy tendons Monocytes and endothelial cells in Achilles tendon	Human	Single-cell RNA sequencing, histology	• Tenocyte A–B: Expression of extracellular tendon microfibrils Tenocyte A Markers: CD10, CD26 and *COL6A3*, *LY6E* Tenocyte B Markers: CD90, *COL4A1*, and *POSTN* • Tenocyte C: Similar to smooth muscle mesenchymal cells Markers: *MYL9*, *ACTA2* • Tenocyte D–E: Similar to fibro-adipogenic progenitors Tenocyte D Markers: *COL6A1*, *COL6A2*, and *COL3A1* Tenocyte E Markers: *TPPP3*, *DCN*, and *FMOD*	Kendal et al., 2020 [51]
Junctional fibroblasts and tendon fibroblasts 1/2 in Achilles tendon	Mouse	Single-cell RNA sequencing, cell trajectory analysis, FACS, histology	• Fibroblast 1: Expression of connective tissue growth factor Markers: *Mkx*, *Spp1* • Fibroblast 2: Differential SPARC Related Modular Calcium Binding 2 (SMOC2) expression Markers: *Dpt*, *Smoc2* • Junctional Fibroblast: Moderate expression of transcripts for collagen types I and Markers: *Col22a1*	Micheli et al., 2020 [65]
Four macrophage clusters termed 1,3,4, and 7 during tendon injury	Mouse	FACS, immunofluorescence, immunohistochemistry, in vivo bioluminescence, single-cell RNA sequencing	• Cluster 1: Tendon resident macrophages Markers: *Cd163*, *Timd4*, *Tgfb1* • Clusters 3 and 4: Expression of M2 markers Markers: CD206 and *Arg1*, *Tgfb1* • Cluster 7: Less expression of M2 markers Markers: *IL-1b*, *Ccl4*, *Erg1*	Sorkin et al., 2020 [66]
Tubulin Polymerization Promoting Protein Family Member 3 (*Tppp3)*^+^ *Pdgfra*^+^ and *Tppp3*^+^*Pdgfra*^−^ subpopulations, macrophages, endothelial cells	Mouse	Immunofluorescence, 10X genomics scRNA-sequencing, FACS, histology, in vitro characterization, qPCR	• *Tppp3*^+^*Pdgfra*^+^ Cells: Can act as tendon stem cells • *Tppp3*^+^*Pdgfra*^−^ Cells: No proliferation and most likely differentiation into Scx^−^ cells found in the healing tendon	Harvey et al., 2019 [67]
Nes^+^ perivascular TSPCs	Human	RNA interference for Nes inhibition, qPCR, microarray analysis	• Increased expression of Nes and expression of tendon-related genes and stem cell markers Markers: Nes, CD146, CD105	Yin et al., 2016 [68]
CD105^+^ and CD105^-^ TSPCs in injured Achilles tendon	Mouse	Histology, immunoblotting	• CD105^-^ cells: Superior chondrogenic potential Markers: Aggrecan, TGF-β1 • CD105^+^ cells: Superior proliferation, important for tendon regeneration Markers: High Scx levels	Asai et al., 2014 [69]
Young and aged TSPCs	Human	FACS, immunohistochemistry, genome-wide microarray, qPCR, senescence analysis, migration experiments	• Young TSPCs: Higher proliferative and migratory capability, multipotent, low F-actin fibers • Aged TSCPs: Less proliferative and migratory, still capable of multilineage differentiation, abundant F-actin fibers, increased senescence, higher ROCK1 activity Markers: microarray identified ca. 1000 differentially expressed genes; in top dysregulated ones, change in genes related to actin dynamics, cell–cell and cell–ECM contact, and migration	Kohler et al., 2013 [54]
TSPCs	Mouse Human	Cell labeling, Western blotting, FACS, qPCR, luciferase reporter assays	• Both mouse and human cells reside mostly in ECM protein-rich regions, with no CD18 expression Mouse Markers: Stem cell antigen marker 1, CD90 Human Markers: CD44, CD90, and CD146	Bi et al., 2007 [70]

**Table 4:** Overview of in vitro studies investigating effects of CPX treatment on tenocytes.

Goals	Cell Types, Treatments, and Assays	Results	Studies
Studying effects of uremic toxins with or without CPX on metabolic activity, vitality, and collagen I expression	• Cells: Human tenocytes isolated from the hamstring tendon • Treatment: Serum-starved tenocytes in 1% FCS for 24 h before incubation with 3, 10, 30, 50, and 100 mg/L with CPX • Assays: Alamar Blue, qPCR for MMP1, IL1β and β1-integrin expression, Western blot for collagen type I detection	CPX suppressed tenocyte activity after short exposure times at higher concentrations after 72 h and this was also observed at therapeutic concentrations. MMP1 mRNA levels increased, and the protein levels of type I collagen decreased. No significant alteration in β1-integrin expression.	Popowski et al., 2020 [27]
To determine the toxicity of LFX on annulus fibrosus cells	• Cells: Rat annulus fibrosis cells • Treatment: LFX with 10, 20, 40, and 80 μg/mL for cell viability and caspase-3 activity assay and 30, 60, and 90 μg/mL for Annexin staining • Assays: Annexin V-FITC/PI staining and caspase-3 activity for apoptosis, MTS for cell viability, Western blot for caspase 3 and MMP3 detection, qPCR for caspase and MMP3 expression levels	LFX concentrations at 30, 60, and 90 μg/mL led to cell apoptosis caused by higher caspase-3 and MMP3 expression and activity.	Bai et al., 2014 [98]
Exploring the impact of CPX on human ligamentocytes in vitro by morphological and molecular methods	• Cells: Cells from the human anterior cruciate ligament • Treatment: 10, 20, and 50 μg/mL CPX for 48 h • Assays: qPCR for collagen I/III, slot blot for Collagen I/II and MMP1, SDS-zymography for MMP activity, fluorescence microscopy for tubulin detection, and cytoskeleton analysis	Collagen mRNA levels were not affected. MMP1 protein levels rose upon 20 μg/mL CPX treatment only. MMP2 and TGF-β1 levels were unmodified. TIMP-1 expression was downregulated. CPX caused no changes in the cytoskeleton.	Menon et al., 2013 [97]
Investigating the alterations in MMP2/9, TIMP1/2, and collagen I expression levels after CPX use	• Cells: Tendon cells from Sprague–Dawley rats • Treatment: 5, 10, 20, or 50 µg/mL CPX for 24 h • Assays: MTT, qPCR for collagen I and MMP2, Western blot for MMP2 and collagen I protein levels, zymography for determining MMP2 and MMP9 levels in medium, reverse zymography for TIMP1/2 levels	CPX reduced viability and increased MMP2 expression and activity in a dose-dependent manner. In contrast, MMP9 expression was not affected. No change in TIMP activity was determined.	Tsai et al., 2011 [99]
Studying the effects of CPX on tenocyte migration	• Cells: Tenocytes from male rat tendons • Treatment: Cells incubated with 5, 10, 20, and 50 μg/mL CPX for 24 h • Assays: Transwells for migration, microscopy to determine tenocyte spreading, Western blot for focal adhesion kinase (FAK) levels and phosphorylation	CPX application led to dose-dependent inhibition of cell growth compared to control. Slow cell migration was possibly due to halted lamellipodium formation in CPX-treated cells. Total FAK expression was not altered, but phosphorylation levels were lowered.	Tsai et al., 2009 [95]
Determining if MOX and CPX cause oxidative stress in vitro in tendon cells, and investigating the protective activity of idebenone (part of the antioxidant MitoQ complex)	• Cells: Tenocytes of normal human Achilles tendons • Treatment: Cells exposed to 0–0.3 mM of CPX or MOX after 24 h • Assays: JC-1 assay for mitochondrial membrane potential, 5-(6)-carboxy-2,7′ dichlorodihydrofluorescein-diacetate for oxidative stress	Increasing concentrations of CPX or MOX damaged the mitochondrial membrane and resulted in decreased membrane potential. Oxidative stress rose after treatment. MitoQ application prevented membrane damage.	Lowes et al., 2009 [100]
Investigating the effect of CPX on the cell cycle of tendon cells and expression of cyclin-dependent kinase (CDK)	• Cells: Rat Achilles tendon cells • Treatment: For MTT assay and cell cycle analysis, 5, 10, 20, or 500 µg/mL CPX was given for 24 h. For immunofluorescence and Western blot: 50 µg/mL CPX for 24 h • Assays: MTT, FACS for cell cycle analysis, PCR for CDK-1, Western blot for (p) CDK1, cyclin B, and checkpoint kinase 1	CPX application lowered cell viability and stopped the cell cycle at the G2/M phase. This is due to condensed chromosomes not properly aligning during replication. Microtubules were also misaligned. (p)CDK-1 and cyclin B expression were lowered.	Tsai et al., 2008 [96]
Examining CPX and IL-1β’s effects on MMP13 expression levels	• Cells: Human Achilles tendon cells • Treatment: 48 h initial incubation with FQs, followed by 48 h supplementation with or without IL-1β together with FQs. • Assays: qPCR for MMP1/13 expression, fluorokine E fluorimetry for MMP1/13 activity measurement	CPX treatment lowered MMP13 expression levels. This effect was stronger when CPX was supplemented together with IL-1β. CPX and NOR enhanced MMP1 expression with or without IL-1β addition. ENR decreased cell count in a dose-dependent manner.	Corps et al., 2005 [101]
Determining ENR-mediated changes on equine superficial digital flexor tendons	• Cells: Cells from superficial digital flexor tendons of foals and older horses • Treatment: Cells exposed to 50 or 100 μg/mL ENR and incubated for 3 days • Assays: Trypan blue for viability, gel zymography for MMP1 expression, TACS Apoptotic DNA laddering for apoptosis, Western and northern blots for decorin and biglycan expression, gas chromatography and mass spectrometry for monosaccharide content determination	Cells from the younger horses were more susceptible to membrane perforations and apoptosis at lower concentrations. No difference in the expression of MMPs, DNA fragmentation, or biglycan mRNA levels was observed. Monosaccharide content decreased by 50%, and decorin amount increased.	Yoon et al., 2004 [102]
Studying the effect of ENR on cell proliferation and proteoglycan synthesis in avian tenocytes	• Cells: Gastrocnemius tendon cells isolated from chicken embryos • Treatment: 25, 50, 100, and 300 μg/mL ENR given for 3 days for proliferation assay. For the apoptosis test, only 50 μg/mL was used. The cells were given 20 or 100 μg/mL ENR for gel zymography • Assays: Trypan blue for cell proliferation measurement, gel zymography for proteolytic activity, Western and northern blotting, electron microscopy, TACS Apoptotic DNA Laddering Kit, Western blotting for decorin detection	Cell number was reduced 3-fold under treatment with 100 and 300 μg ENR. ENR led to the formation of tightly woven collagen fibrils in the ECM. Decorin mRNA levels were lowered after 72 h, and protein levels decreased dose-dependently. Monosaccharide levels were lower and composition was also altered.	Yoon et al., 2004 [103]
Investigating the genotoxicity potential and mechanism of PFX, LFX, CPX, and OFX	• Cells: Spontaneously immortalized clones of tenocytes from rabbit Achilles tendon • Treatment: 0.01 μM, 0.1 μM, 1 μM, 10 μM, 0.1 mM, and 1 mM FQs given for 24 h or 72 h • Assays: Neutral red, AlamarBlue for determining redox status, monobromobimane as glutathione test	All FQs were mildly toxic to tenocytes, the toxicity was higher after 72 h. CPX and PFX treatment decreased cell viability and redox status in the highest concentration after 72 h. Meanwhile, ROS levels increased at higher concentrations (>10 μM CPX). OFX and LFX caused a mild redox activity decrease.	Pouzaud et al., 2004 [104]
Exploring the effects of CPX on signaling responses of tendon cells	• Cells: Tendon cells from a chronic human Achilles tendinopathy sample • Treatment: Initial application of 50 µg/mL CPX, afterwards 1 ng/mL IL.1ß was given to cells After 2 days, medium supplemented with either IL-1β, CPX, or both • Assays: Prostaglandin E2 ELISA, Western blot for cyclooxygenase-2 levels	IL-1β treatment heightened PGE expression, whilst CPX lowered it. In contrast, CPX did not affect the cyclooxygenase-2 expression levels even when IL-1β was present.	Corps et al., 2003 [105]
Studying the impact of different CPX concentrations on Achilles tendon/paratenon and shoulder capsule fibroblast function	• Cells: Fibroblasts from canine Achilles tendon, Achilles paratenon, and shoulder capsule • Treatment: 5 μg/mL, 10 μg/mL, or 50 μg/mL CPX • Assays: MTT and Coulter cell counter for cell number determination, H-proline incorporation for collagen synthesis, sulfate incorporation for proteoglycan measurement, radiolabeled casein for matrix degradation quantification	CPX supplementation caused increased matrix-degrading activity, decreased matrix synthesis, and reduced cell proliferation in each cell population. Collagen synthesis was significantly lowered. CPX was most toxic at 50 μg/mL concentration.	Williams et al., 2000 [106]
Determining the influence of FQs on tendon cells from various species and describing age-dependency	• Cells: Human Achilles tendon samples (ages 3 months to 79 years), dogs, mini-pigs, rats, marmosets • Treatment: 3 regimens (all 72 h duration): FQ+ triamcinolone acetonide (a common corticosteroid used for skin conditions), FQ treatment, then 3 days rest • Assays: Neutral red and MTT for cell viability, 5-bromo-2′-deoxyuridine for cell proliferation, collagen I immunofluorescence	CPX-treated cells showed no age or species-specific effects. The cytotoxicity of CPX was increased when combined with corticosteroids.	Kempka et al., 1996 [107]

## Abbreviations

CPX	Ciprofloxacin
CD	Cluster of differentiation
CDK	Cyclin-dependent kinase
CSF1	Colony stimulating factor 1
CX3CL1	C-X3-C motif chemokine ligand 1
CX3CR1	C-X3-C motif chemokine receptor 1
ECM	Extracellular matrix
EGR	Early growth response factor
EMA	European Medicines Agency
ENR	Enrofloxacin
FACS	Flow cytometry
FAK	Focal adhesion kinase
FDA	Food and Drug Administration
FGF	Fibroblast Growth Factor
FLX	Fleroxacin
FQ	Fluoroquinolone
FQAD	Fluoroquinolone-associated disability
IL	Interleukin
LFX	Levofloxacin
MMP	Matrix metalloproteinase
Mrp4	Multidrug resistance protein 4
MSC	Mesenchymal stem cell
Mkx	Mohawk
MOX	Moxifloxacin
Nes	Nestin
NOR	Norfloxacin
PFX	Pefloxacin
PDGF	Platelet-derived growth factor
PDGFRA	Platelet-derived growth factor receptor alpha
qPCR	Quantitative real-time PCR
ROS	Reactive oxygen species
Scx	Scleraxis
SLRP	Small Leucin-Rich Proteoglycans
SMOC	SPARC Related Modular Calcium Binding
SLC	Solute carrier family 40 member 1
SPX	Sparfloxacin
TSPC	Tendon stem/progenitor cells
TNMD	Tenomodulin
TGF	Transforming growth factor
Tppp3	Tubulin Polymerization Promoting Protein Family Member 3
